# Evaluation of the optimal cooling temperature for the face measured by the tissue perfusion during hilotherapy using laser Doppler spectrophotometry

**DOI:** 10.1038/s41598-021-89313-1

**Published:** 2021-05-07

**Authors:** Florian Peters, Nicole Heussen, Jana Herbstmann, Stephan Christian Möhlhenrich, Anna Bock, Kristian Kniha, Frank Hölzle, Ali Modabber

**Affiliations:** 1 Department of Oral, Maxillofacial and Facial Plastic Surgery, School of Medicine, University Hospital RWTH Aachen, Pauwelsstr. 30, 52074 Aachen, Germany; 2 Department of Medical Statistics, University Hospital RWTH Aachen, Pauwelsstr. 30, 52074 Aachen, Germany; 3 Department of Orthodontics, University Witten/Herdecke, Private Universität Witten/Herdecke GmbH, Alfred-Herrhausen-Straße 45, 58448 Witten, Germany

**Keywords:** Rehabilitation, Surgery

## Abstract

After craniofacial trauma, symptoms like swelling and pain occur. Cooling reduces these symptoms but the optimal cooling temperature for a maximum benefit without adverse effects is unclear. 30 participants were cooled at 10 °C, 15 °C, 20 °C, 25 °C and 30 °C for 30 min. Before cooling and at 15, 30, 45 and 60 min after cooling, the skin blood flow, oxygen saturation (SO) and haemoglobin concentration (Hb) were measured by laser Doppler spectrophotometry at 2 mm and 8 mm depth. The skin temperature was measured, and the participant’s satisfaction was marked on a visual analogue scale. There were significant differences between males and females in the blood flow, SO and Hb (*p* < 0.0001). After cooling, the blood flow, SO and Hb was reduced. The measured values rose slightly above the initial values 60 min after cooling. Depending on the cooling temperature the decrease in blood flow, SO and Hb was significantly different. Both sexes were most comfortable with a 25 °C cooling temperature and satisfaction decreased with lower temperatures. Significant differences for the satisfaction between both sexes were measured (10 °C: *p* < 0.0001, 15 °C: *p* < 0.0001, 20 °C: *p* = 0.0168, 25 °C: *p* = 0.0293). After 60 min, the males and females exhibited mild skin hyperthermia. The optimal cooling temperatures their physiological effects and their perception for females and males were different. For females, around 20 °C is an optimal cooling temperature. For males, it is around 15–20 °C.

## Introduction

Craniofacial trauma is common. Most cases result from everyday activities, followed by sport accidents and assaults. Approximately 62.5% of patients suffer soft tissue injuries, and 37.5% suffer fractures^1^. Actually after treatment, inflammation and swelling occurs. Trauma leads to inflammation in the body tissues. Tissue damage leads to the release of messenger substances that result in the immigration of immune cells. These cells release cytokines, which lead to capillary leakage. Through a capillary leak, more intravascular fluid penetrates the tissue, leading to swelling^2^. Tissue swelling increases the diffusion distance for oxygen to the cells. A lack of oxygen occurs, culminating in an intensification of the inflammatory reaction^3^. For this reason, swelling of traumatized tissue should be prevented.

Since ancient times, cold in the form of water, ice or snow have been applied to inflamed areas of the body^4^. The cooling of inflamed skin has a positive effect on swelling and pain. The HILOTHERM Clinic (Hilotherm GmbH, Argenbühl-Eisenharz, Germany) facilitates the continuous cooling of patients directly after surgical treatment. It has been compared to cold compresses and has been found to be effective for the reduction of swelling and pain^5–8^. One reason is the reduced distribution of cytokines like prostaglandin E_2_, which is known to be a marker for inflammation^9^. This reaction reduces the inflammatory reaction and the resulting capillary leak. Another reason for a reduction of swelling is the reduced blood flow during cooling. When less blood flows through the tissue^10,11^, less blood plasma can leak through the capillary leak and fewer immune cells enter the tissue.

Several cooling temperatures have been reported in the literature^12,13^. The effects of cooling temperatures on tissues are different for males and females^14,15^. In females the blood flow was reduced greater than in males when cooling with the same temperature. Studies have indicated that the optimal cooling temperature after intraoral surgery is 10–15 °C^16^.

While attempts are being made to reduce swelling, the healing of damaged tissues should not be interrupted by cryotherapy. Oxygen is crucial for wound healing and to maintain the metabolism of the cells. To provide sufficient oxygen to the tissue, the oxygen saturation (SO_2_) and haemoglobin concentration (Hb) should not drop due to cooling^17,18^. However, since cooling also causes a decrease in metabolism in the cooled tissue^9^, a small decrease in SO_2_ and Hbs can be tolerated by the healing tissue. Therefore, the blood flow should be reduced, and the SO_2_ and the Hb should be kept stable after cooling. Reactive hyperemia has been described in the literature after cooling^19^. During reactive hyperemia, there is a higher blood flow, which could increase the swelling. Therefore, the reactive hyperemia should be as low as possible.

The aim of this study was to determine the possibility of an optimal cooling temperature reducing blood flow while maintaining constant SO_2_ and Hbs levels in the cooled facial tissues. The secondary outcome was the participants’ satisfaction with the temperatures that were used. The hypothesis is that males and females need a different cooling temperature for achieving optimal physiological and satisfaction values.

## Methods

### Participants

**Figure 1:**
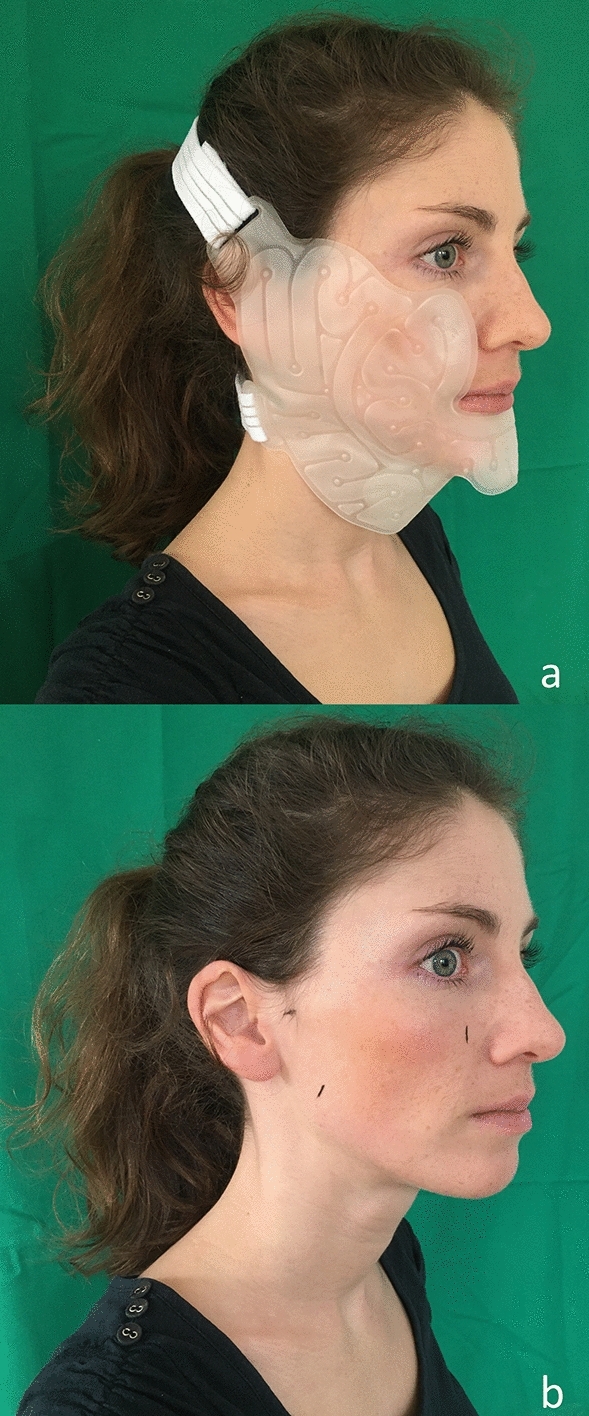
(**a**) Hilotherm cooling mask fixed on volunteer. (**b**) Measuring points marked to volunteer’s face.

Institutional approval was granted by the ethics committee of Aachen University Hospital (EK 077/77). All procedures performed in this study were in accordance with the 1964 Helsinki Declaration and its later amendments or comparable ethical standards. Informed consent from the participant in Fig. 1 was obtained for publishing the images in an online open access publication. Thirty participants were recruited, and each provided written informed consent prior to participation in the study. The sample comprised 15 females and 15 males with a mean age of 24.3 years. The inclusion criteria were as follows: the participants needed to be older than 18 years, healthy, unmedicated and non-smoking; in addition, they could not have had prior facial surgeries or injuries. Volunteers were asked specifically about reactions to cold such as Raynaud's syndrome. All volunteers reported normal reactions to cold with no physiological abnormalities. Beards had to be shaved to prevent the hair from interfering with the measurement method.

### HILOTHERM Clinic HT02

The HILOTHERM Clinic HT02 comprises a cooling unit, a water tank and a pump. The tank was filled with demineralized water that was cooled to adjustable temperatures of + 10 °C to + 30 °C. The HILOTHERM Clinic was connected by hoses to a pre-shaped cooling mask that was suitable for the lower face. The mask was fitted to the volunteer’s face to ensure contact between the mask and the skin with the strap of the mask and adhesive tape. The device continuously pumped the water through the mask, which was affixed to the participant’s face (Fig. 1a).

### O2C (Oxygen to See)

The O_2_C (Oxygen to See; LEA Co. Gießen, Germany) is a spectrophotometer that measures the blood flow, SO_2_ and Hb in the tissues close to the skin surface (2 mm) and at a greater depth (8 mm). The probe (LF-2) connected to a glass-fibre cable was attached to the device. The SO_2_ and Hb were measured by the backscatter of the emitted light with 500–630 nm. Depending on the amount of hemoglobin and the saturation of its oxygen binding sites the emitted light is absorbed or reflected. The spectrum of the backscatter was used to calculate both values. The Hb and SO_2_ were the mean values of the entire measured tissue that was penetrated by the light. The measured SO_2_ and Hb are not comparable to the arterial SO_2_. The measured values are averaged values from the Hb and SO_2_ in the tissue. The O_2_C measures the Hb and SO_2_ in the venous-capillary blood vessels. The emitted light gets totally absorbed from vessels bigger than 100 µm^20^. Therefore, the measured values from the O_2_C represent the local conditions in the measured tissues and not the global values. Additionally, a laser Doppler velocimetry was performed. Laser light with the wavelength of 830 nm was emitted and the backscatter was received. The laser light with a frequency of 830 nm hits the moving erythrocytes in the small vessels. The relative movement of the erythrocytes in relation to the measurement probe results in a change in the wavelength of the reflected light. The changes in the wavelength of the backscatter allowed for the calculation of blood flow^21,22^. The O_2_C measured the flow and Hb in arbitrary units (AUs). The SO_2_ was displayed in percent. The percentage displays the amount of binding sites of the hemoglobin in the bloodstream that are occupied by oxygen. The accuracy of measuring is 1 AU for flow an Hb respectively 1% for the SO_2_.

### Study protocol

Cooling was performed at a randomized sequence of five temperatures: 10 °C, 15 °C, 20 °C, 25 °C and 30 °C. A minimum of 24 h passed between cooling at a different temperature. Prior to each session, the participant had to lie down for 30 min without any activity that would increase circulation. Then, a baseline measurement was taken for blood flow, SO_2_, Hb and skin temperature. For the O_2_C measurements, the probe was applied to three different measuring sites, which were chosen because them being the likely sites for swelling after a facelift or surgery on the zygomatic bone, mandible, teeth, or orbit^5,7,13,23^. The measurement site on the masseteric muscle showed the effects on the skin and muscular tissues, and the other sites showed the effects on relatively thin skin with underlying bone. The first measuring site was 1 cm frontal to the Tragus of the ear, the second was the midsection of the masseter muscle, and the third was 2 cm lateral to the nasal wing (Fig. 1b). The skin temperature was measured at the same three measurement sites. Every measurement, including the baseline measurement, was performed twice in quick succession, and the mean value was taken to reduce measurement errors. While measuring the O_2_C device averages the measured values over a period of 10 s.

For clarity reasons and because each participant was measured at the same measurement sites the mean value from the three measurement points was used for further statistical evaluation.

A touchless infrared thermometer (Reer GmbH, Leonberg, Germany) was used to determine the skin temperature. This device focuses the infrared radiation emitted by the skin on a detector. The detector converts the infrared radiation into electrical signals that can be displayed as numerical values^24^. The accuracy of this thermometer was ± 0.2 °C according to the user guide. To prevent microcirculatory disturbances due to compression of the capillaries, the measurement was performed touchless without applying pressure to the skin. Thereafter, the mask was affixed to the participant’s face and cooled with the designated temperature for 30 min. It was then removed, and the skin temperature as well as the O_2_C parameters were measured as before at the previously marked measurement sites. Consecutive measurements were performed after 15, 30, 45 and 60 min. During the entire session, the participants were not allowed to stand up to avoid an increase in circulation.

After the measurement session, the participants were asked to indicate their satisfaction with the cooling temperature on a visual analogue scale (VAS) of 1 to 10, where 1 was extreme discomfort and 10 was extreme comfort.

### Statistical evaluation

To describe the evolution of the blood flow, SO_2_ and Hb respectively at the various temperatures and positions, a linear mixed-effects model with random slope and intercept with an unstructured covariance structure was fitted to the data. The sex was included as an additional fixed factor in the model. Pairwise comparisons of the various temperatures for males and females were evaluated by the corresponding linear contrasts. The model assumptions and fit were assessed by visual inspection of residuals, and influence diagnostics. The males’ and females’ VAS satisfaction scores for each temperature were compared through the Mann–Whitney *U* test. For all the comparisons, the significance level was set to 5% because of the exploratory nature of the investigation. No adjustment was made to the significance level. The results are reported as means and standard deviations (± SD); and two-sided *p*-values. All analyses were performed trough SAS version 9.4 (PROC MIXED), SAS Institute Inc., Cary, NC, USA.

### Ethics approval and consent to participate

Institutional approval was granted by the ethics committee of Aachen University Hospital (EK 077/77). Informed consent was obtained from every participant to take part in the present study. All procedures performed in this study were in accordance with the 1964 Helsinki Declaration and its later amendments or comparable ethical standards.

### Consent for publication

Informed consent for publication of Fig. 1 was obtained from the participant in written form.

## Results

The values acquired from the three different measurement sites in the volunteers faces differed significantly (All measured values *p* < 0.0001; besides Hb deep *p* = 0.0002; SO_2_ deep *p* = 0.0024).

### Blood flow

**Figure 2:**
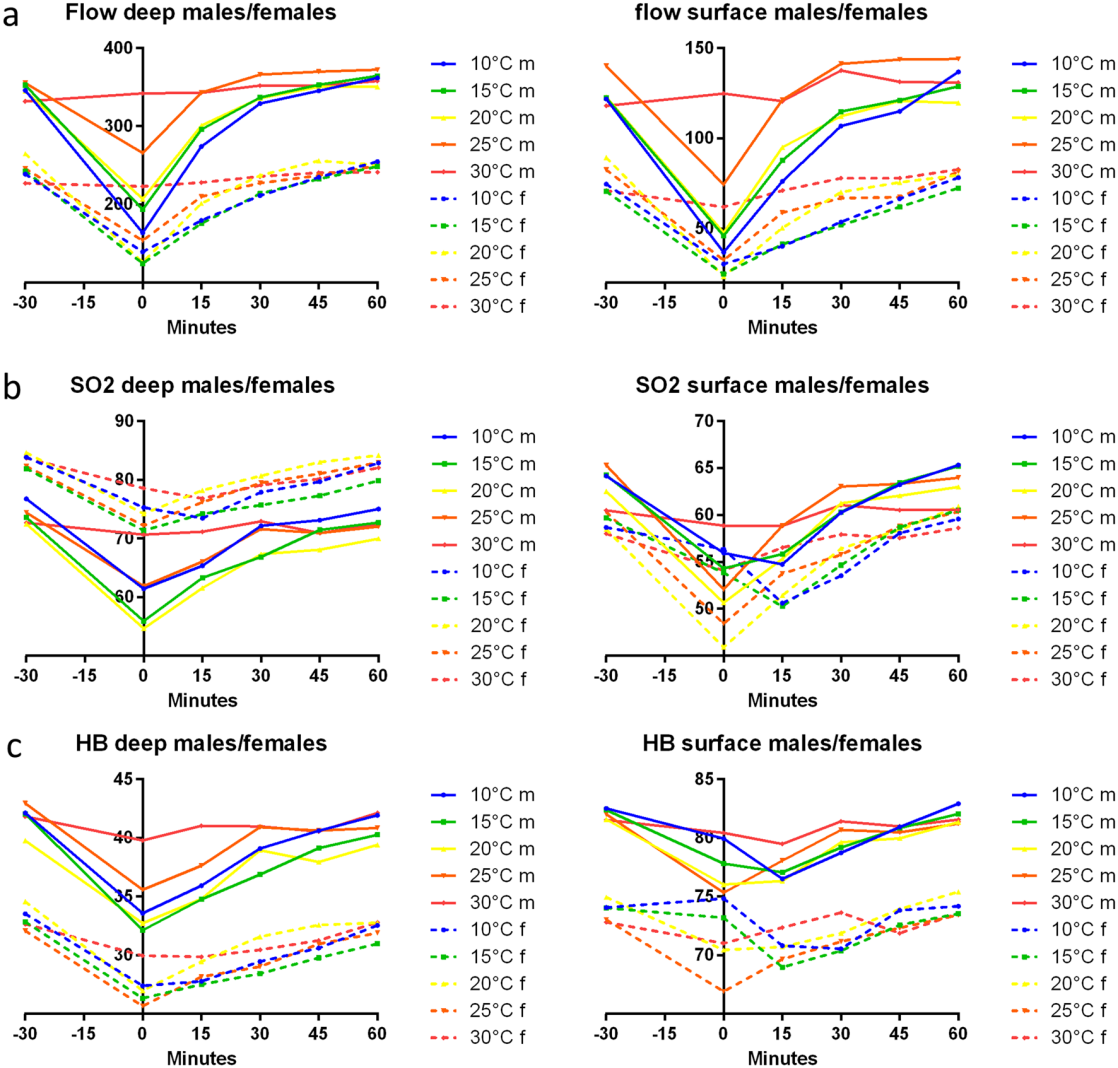
Progress of measured values before and after cooling: (**a**) surface and deep tissue blood flow [AU] in females/males, (**b**) surface and deep tissue oxygen saturation [%] in females/males, (**c**) surface and deep tissue haemoglobin concentration [AU] in females/males.

The blood flow in female participants was reduced immediately after cooling for each temperature to the baseline (Fig. 2a). The surface blood flow after cooling at 10 °C, 15 °C, 20 °C and 25 °C was significantly lower than at 30 °C. There were rarely no significant differences in the blood flow in the deep tissues at cooling with 10 °C, 15 °C, 20 °C and 25 °C (Table 1). The greatest reduction occurred after cooling at 15 °C from initially 243.0 ± 90.17 AU to 124.7 ± 42.67 AU immediately after cooling and cooling with 20 °C resulting in a reduction from 264.8 ± 100.3 AU to 126.2 ± 41.08 AU immediately after cooling (Fig. 2a). After 60 min, every cooling temperature besides 20 °C and the surface flow at 25 °C resulted in reactive hyperemia compared to the baseline value (Table 2). This increase was not statistically significant. At 60 min, cooling at 20 °C resulted in a decrease in blood flow of 12.4 AU in the deep tissues and 9.66 AU at the surface compared to the measured baseline (Table 2). The surface flow decreased by 0.96 AU after cooling at 25 °C.

**Table 1:** *p*-values of blood flow immediately after cooling at different temperatures.

Female/deep	10 °C	15 °C	20 °C	25 °C	30 °C
10 °C		0.0647	0.0031	0.2355	< 0.0001
15 °C	0.0647		0.2632	0.0024	< 0.0001
20 °C	0.0031	0.2632		< 0.0001	< 0.0001
25 °C	0.2355	0.0024	< 0.0001		< 0.0001
30 °C	< 0.0001	< 0.0001	< 0.0001	< 0.0001	

Male/deep	10 °C	15 °C	20 °C	25 °C	30 °C
10 °C		0.0043	< 0.0001	< 0.0001	< 0.0001
15 °C	0.0043		0.0537	< 0.0001	< 0.0001
20 °C	< 0.0001	0.0537		< 0.0001	< 0.0001
25 °C	< 0.0001	< 0.0001	< 0.0001		< 0.0001
30 °C	< 0.0001	< 0.0001	< 0.0001	< 0.0001	

Female/surface	10 °C	15 °C	20 °C	25 °C	30 °C
10 °C		0.6533	0.0727	0.8313	< 0.0001
15 °C	0.6533		0.1784	0.8134	< 0.0001
20 °C	0.0727	0.1784		0.1135	< 0.0001
25 °C	0.8313	0.8134	0.1135		< 0.0001
30 °C	< 0.0001	< 0.0001	< 0.0001	< 0.0001	

Male/surface	10 °C	15 °C	20 °C	25 °C	30 °C
10 °C		0.254	0.1796	0.0002	< 0.0001
15 °C	0.254		0.8405	0.0099	< 0.0001
20 °C	0.1796	0.8405		0.0175	< 0.0001
25 °C	0.0002	0.0099	0.0175		< 0.0001
30 °C	< 0.0001	< 0.0001	< 0.0001	< 0.0001

**Table 2:** *p*-values of blood flow 60 min after cooling at different temperatures and mean flow values before cooling and 60 min after cooling.

Females/deep	10 °C	15 °C	20 °C	25 °C	30 °C	− 30 min	60 min	Difference
10 °C		0.366	0.0502	0.332	0.4347	238.86	254.75	15.89
15 °C	0.366		0.2906	0.9473	0.9024	242.98	248.72	5.74
20 °C	0.0502	0.2906		0.3216	0.2392	264.78	252.38	− 12.4
25 °C	0.332	0.9473	0.3216		0.8504	245.75	249.6	3.85
30 °C	0.4347	0.9024	0.2392	0.8504		227.11	241.34	14.23

Males/deep	10 °C	15 °C	20 °C	25 °C	30 °C	− 30 min	60 min	Difference
10 °C		0.899	0.2203	0.5454	0.7009	345.82	361.8	15.98
15 °C	0.899		0.2718	0.4644	0.6096	352.65	364.3	11.65
20 °C	0.2203	0.2718		0.0673	0.1076	345.21	350.3	5.09
25 °C	0.5454	0.4644	0.0673		0.8257	335.31	372.38	37.07
30 °C	0.7009	0.6096	0.1076	0.8257		331.82	357.81	25.99

Females/surface	10 °C	15 °C	20 °C	25 °C	30 °C	− 30 min	60 min	Difference
10 °C		0.6358	0.465	0.9551	0.4083	74.67	78.2	3.53
15 °C	0.6358		0.797	0.6766	0.1935	70.54	72.54	2
20 °C	0.465	0.797		0.4999	0.1197	89.28	79.62	− 9.66
25 °C	0.9551	0.6766	0.4999		0.3773	82.54	81.58	− 0.96
30 °C	0.4083	0.1935	0.1197	0.3773		70.97	82.8	11.83

Males/surface	10 °C	15 °C	20 °C	25 °C	30 °C	− 30 min	60 min	Difference
10 °C		0.2807	0.0221	0.8209	0.5934	121.74	136.81	15.07
15 °C	0.2807		0.2256	0.3944	0.5858	122.44	128.81	6.37
20 °C	0.0221	0.2256		0.0392	0.0791	122.81	119.62	− 3.19
25 °C	0.8209	0.3944	0.0392		0.7588	140.1	143.94	3.84
30 °C	0.5934	0.5858	0.0791	0.7588		117.96	130.86	12.9

Immediately after cooling at 10 °C, 15 °C, 20 °C and 25 °C, the males exhibited reduced blood flow (Fig. 2a). Cooling at 30 °C led to an increase. Immediately after cooling, there were statistically significant differences in the blood flow values for almost every temperature. The exception were the deep tissue blood flow values at 20 °C and 15 °C. A significant difference in the flow values was measured at the surface immediately after cooling at 25 °C and 30 °C (Table 1). The lowest post-cooling blood flow was achieved with 10 °C. For each cooling temperature, a difference between the initial and resulting flow values after 60 min existed. An increase in blood flow and thus reactive hyperemia was recorded for every temperature except 20 °C (Table 2). The smallest increase in blood flow, 5.09 AU in the deep tissues, and even a decrease of 3.19 AU at the surface were achieved with the 20 °C cooling temperature (Table 2).

### Saturation of oxygen

The females’ post-cooling SO_2_ values for each temperature were lower than the initial values (Fig. 2b). The lowest SO_2_ values achieved with 10 °C and 30 °C were those for the deep tissues 15 min after cooling. At the surface, the deepest SO_2_ values were measured at 15 min after cooling at 10 and 15 °C. A significant difference between the lowest SO_2_ values in the deep tissues were observed for 15 °C and 30 °C (*p* = 0.0269). There were statistically significant differences in the lowest SO_2_ values for each temperature measured at the surface (Table 3).

**Table 3:** *p*-values of the lowest SO_2_ values after cooling at different temperatures.

Females/deep	10 °C	15 °C	20 °C	25 °C	30 °C
10 °C		0.5811	0.8538	0.8107	0.0964
15 °C	0.5811		0.4618	0.7547	0.0269
20 °C	0.8538	0.4618		0.6718	0.1393
25 °C	0.8107	0.7547	0.6718		0.0572
30 °C	0.0964	0.0269	0.1393	0.0572	

Males/deep	10 °C	15 °C	20 °C	25 °C	30 °C
10 °C		0.0583	0.0252	0.4387	< 0.0001
15 °C	0.0583		0.7293	0.0076	< 0.0001
20 °C	0.0252	0.7293		0.0026	< 0.0001
25 °C	0.4387	0.0076	0.0026		< 0.0001
30 °C	< 0.0001	< 0.0001	< 0.0001	< 0.0001	

Females/surface	10 °C	15 °C	20 °C	25 °C	30 °C
10 °C		0.5565	0.001	0.0353	0.0069
15 °C	0.5565		0.0071	0.129	0.001
20 °C	0.001	0.0071		0.239	< 0.0001
25 °C	0.0353	0.129	0.239		< 0.0001
30 °C	0.0069	0.001	< 0.0001	< 0.0001	

Males/surface	10 °C	15 °C	20 °C	25 °C	30 °C
10 °C		0.2299	0.0013	0.0018	0.001
15 °C	0.2299		0.0426	0.0536	< 0.0001
20 °C	0.0013	0.0426		0.9217	< 0.0001
25 °C	0.0018	0.0536	0.9217		< 0.0001
30 °C	0.001	< 0.0001	< 0.0001	< 0.0001	

The males’ post-cooling SO_2_ values for each temperature were lower than the initial values (Fig. 2b).

### Haemoglobin concentration

In the females, cooling at each temperature led to lower Hb than those initially measured (Fig. 2c). Immediately after cooling at 10 °C, 15 °C, 20 °C and 25 °C, there were no statistically significant differences in the deep tissue Hbs (Table 4). The deep tissue values indicated that the Hbs immediately after cooling at 30 °C were significantly higher than those observed at the lower temperatures. The Hb values after cooling at 25 °C were significantly lower than those at all the other temperatures except 15 °C (Table 4). The lowest Hb values at the surface were not obtained directly after cooling for any of the cooling temperatures. The lowest Hb value for 10 °C was recorded 30 min after cooling, and for 15 °C, it was 15 min after cooling.

**Table 4:** *p*-values of the lowest haemoglobin concentration values after cooling at different temperatures.

Females/deep	10 °C	15 °C	20 °C	25 °C	30 °C
10 °C		0.5038	0.4025	0.3542	0.0072
15 °C	0.5038		0.8661	0.7963	0.0008
20 °C	0.4025	0.8661		0.9288	0.0004
25 °C	0.3542	0.7963	0.9288		0.0003
30 °C	0.0072	0.0008	0.0004	0.0003	

Males/deep	10 °C	15 °C	20 °C	25 °C	30 °C
10 °C		0.1973	0.793	0.1494	< 0.0001
15 °C	0.1973		0.121	0.0064	< 0.0001
20 °C	0.793	0.121		0.239	< 0.0001
25 °C	0.1494	0.0064	0.239		< 0.0001
30 °C	< 0.0001	< 0.0001	< 0.0001	< 0.0001	

Females/surface	10 °C	15 °C	20 °C	25 °C	30 °C
10 °C		0.1094	0.5551	0.0013	0.2393
15 °C	0.1094		0.3121	0.1064	0.0055
20 °C	0.5551	0.3121		0.0087	0.0775
25 °C	0.0013	0.1064	0.0087		< 0.0001
30 °C	0.2393	0.0055	0.0775	< 0.0001	

Males/surface	10 °C	15 °C	20 °C	25 °C	30 °C
10 °C		0.5252	0.9943	0.3471	0.0003
15 °C	0.5252		0.5299	0.1152	0.0026
20 °C	0.9943	0.5299		0.3434	0.0003
25 °C	0.3471	0.1152	0.3434		< 0.0001
30 °C	0.0003	0.0026	0.0003	< 0.0001	

The males’ post-cooling Hbs for each temperature were lower than the initial values (Fig. 2c). The lowest Hbs after cooling at 30 °C were significantly different from those achieved at the other temperatures (Table 4). The lowest post-cooling surface Hbs at 10 °C, 15 °C and 30 °C were measured at 15 min after each application.

### Satisfaction with cooling

**Figure 3:**
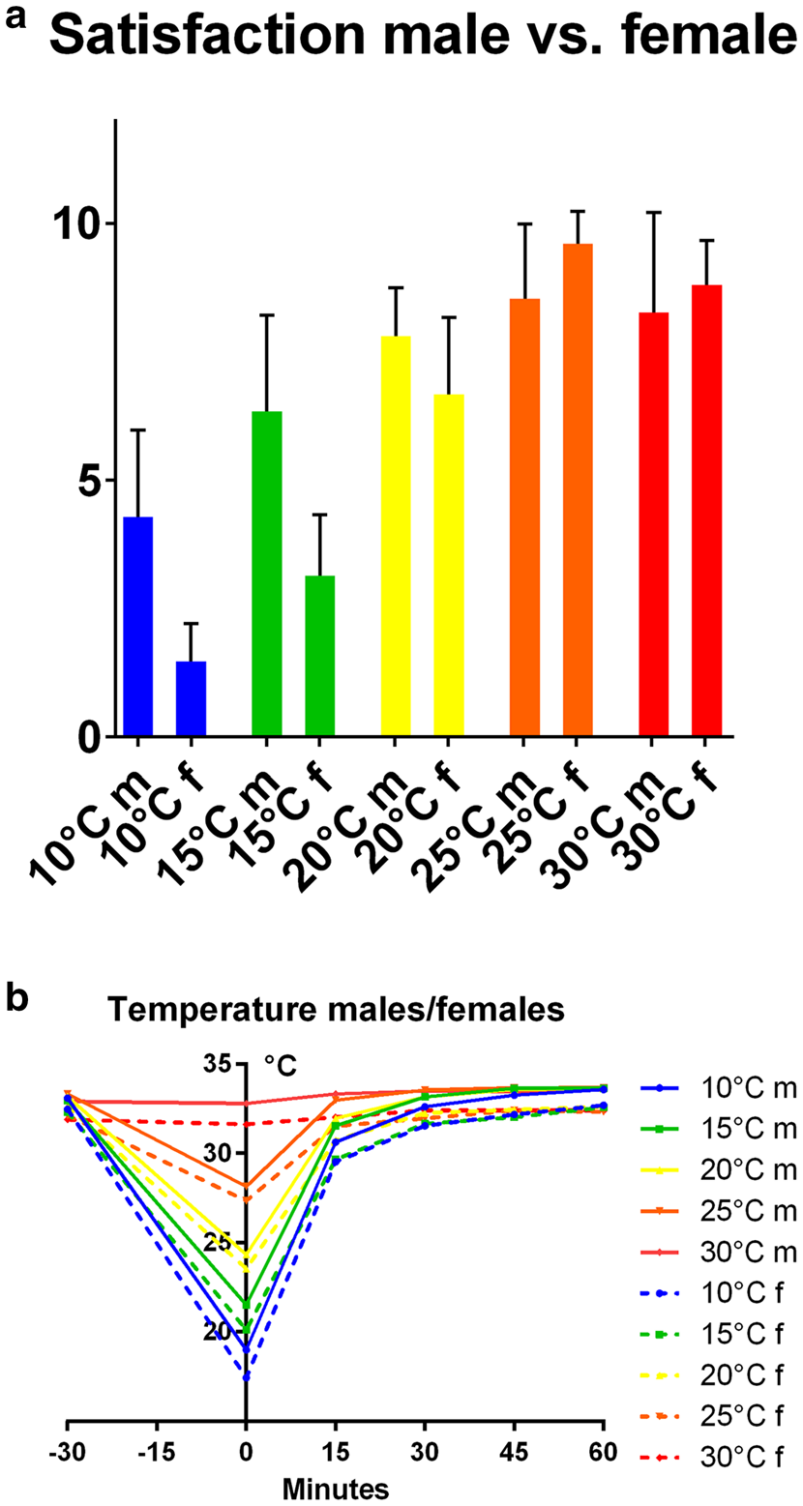
(**a**) Participants’ satisfaction with cooling temperatures, (**b**) females’/males’ measured skin temperature [°C].

The participants’ satisfactions showed statistically significant differences in the females’ satisfaction levels (*p* < 0.0001, 30 °C vs. 25 °C: *p* = 0.0147; Fig. 3a; Table 5). The significant differences in the males’ satisfaction values for the cooling temperatures were observed for 10 °C vs. 15 °C and 15 °C vs. 20 °C (10 °C vs. 15 °C: *p* = 0.0072, 15 °C vs. 20 °C: *p* = 0.0131; Table 5).

**Table 5:** Absolute satisfaction values with cooling temperatures and surface skin temperature of males and females.

Females	10 °C	15 °C	20 °C	25 °C	30 °C
	1.467 ± 0.743	3.133 ± 1.187	6.667 ± 1.496	9.6 ± 0.6325	8.8 ± 0.8619
− 30 min	32.48 °C	32.33 °C	32.62 °C	32.13 °C	31.90 °C
+ 60 min	32.69 °C	32.58 °C	32.61 °C	32.33 °C	32.39 °C
Difference	0.21 °C	0.25 °C	− 0.01 °C	0.20 °C	0.49 °C

Males	10 °C	15 °C	20 °C	25 °C	30 °C
	4.286 ± 1.684	6.333 ± 1.877	7.8 ± 0.941	8.533 ± 1.457	8.267 ± 1.944
− 30 min	33.10 °C	32.95 °C	33.18 °C	33.32 °C	32.90 °C
+ 60 min	33.58 °C	33.64 °C	33.65 °C	33.68 °C	33.57 °C
Difference	0.48 °C	0.69 °C	0.47 °C	0.36 °C	0.67 °C

Both sexes were most comfortable with 25 °C cooling temperature and satisfaction decreased with lower temperatures. The decrease in satisfaction in the females was greater than that in males (Table 5, Fig. 3a). Between both sexes, there were significant differences in the satisfaction at the 10 °C, 15 °C, 20 °C and 25 °C temperatures (10 °C: *p* < 0.0001, 15 °C: *p* < 0.0001, 20 °C: *p* = 0.0168, 25 °C: *p* = 0.0293). The difference in the satisfaction values for 30 °C was not significant (*p* = 0.5439).

### Skin temperatures during cooling sessions

The lower the cooling temperatures, the lower were the skin temperatures. Immediately after cooling, the skin temperature was significantly different for each cooling temperature (*p* < 0.0001). Within 30 min of the end of each cooling session, the skin temperatures rose such that they were close to the initial values. After 60 min, the males and females exhibited hyperthermia. The exception were the females with cooling at 20 °C (Fig. 3b; Table 5).

## Discussion

Swelling is one of the major patient complaints after trauma or surgery^25^. Medications, such as ibuprofen and cortisone, can be used to reduce pain and swelling. However, severe adverse effects must be considered when medications are being used to decrease swelling. Cooling like Hilotherapy is a comfortable and appropriate technique for providing long-term cooling at a defined cooling temperature^5–7,26^, and it is tolerated well by patients^13^.

Previous studies have indicated that cooling, helps to reduce localized inflammatory symptoms^27–30^. However, the appropriate temperature for reducing swelling, pain and other symptoms of inflammation without harming the patient is unclear. Using the wrong temperature could even increase inflammation and the accompanying symptoms^31^. A cooling temperature below 10 °C could result in tissue damage^32–34^. If the temperature is too high, the effect is minimal.

This study used two modern devices: the HILOTHERM Clinic HT02, which provides steady cooling at adjustable temperatures^35^, and the O_2_C, which provides non-invasive measurements of tissue perfusion. The O_2_C device is commonly used for medical purposes^21,36,37^.

To determine the optimal cooling temperature, the development of blood flow, the Hb and the SO_2_ were measured. Soft tissue perfusion is reduced during cooling^10,11^, and the SO_2_ and Hb in the tissues also decrease^38–40^. The reduction of blood flow results in less swelling. However, oxygen is crucial for wound healing. This requires that a high SO_2_ is maintained in the tissues during healing^17,18^. Another effect of cooling is the reduced metabolism in the cooled tissues^9^. A slight decrease in the SO_2_ and Hb can be tolerated by the healing tissues. After cooling, the blood perfusion rises again, and hyperaemia can occur. Hyperaemia should be avoided to prevent an exaggerated increase in swelling and inflammation after cooling. The present study found that the best temperature for facial cooling was preferably one that allowed for a significant reduction in blood flow combined with the maintenance of the SO_2_ and HB at the initial values. In addition, satisfaction with the cooling was considered because if patient satisfaction is too low the cooling will not be applied by the patient.

There were significant differences in the values for the sexes (*p* < 0.0001; SO_2_ deep *p* = 0.0183; Hb surface *p* = 0.001). The exception was the SO_2_ measured at the surface (*p* = 0.1736). That the optimal cooling temperature for females and males are different cannot be ruled out. These results confirm those of previous studies that found significant differences in the reduction of blood flow in males and females. Therefore, the results for the sexes were analysed separately. These studies even found that the response to cooling varied in the menstrual cycle^14,15^. In the present study, the female participants’ menstrual cycles were not considered; thus, whether all the phases of the menstrual cycle were represented is unknown. The female volunteers in this study had a random mixture of menstrual cycle phases. This random mix of menstrual cycle phases is also expected in the later female patient group to be cooled. Therefore, the random mixture of menstrual cycle phases in the group of female volunteers in this study is no disadvantage.

### Females

In the females, the greatest reduction in blood flow occurred with the 15 °C and 20 °C temperatures. Immediately after cooling, there was no significant difference between these blood flow values (surface: *p* = 0.1784; deep: *p* = 0.2632). Only the 20 °C temperature did not lead to an increase in blood flow at either measuring depth compared to the baseline value (Table 2). All other cooling temperatures resulted in a mild reactive hyperaemia after 60 min after cooling. The SO_2_ was reduced after cooling at each temperature. In the deep tissue measurements, the lowest SO_2_ values after cooling at 20 °C were not significantly lower than those at any other temperature (Table 3). At the surface, the lowest SO_2_ after cooling at 20 °C was significantly lower than that at the other temperatures except 25 °C (Table 3). Every temperature led to a decrease in the Hb. Cooling at 20 °C did not lead to significantly lower Hb values measured in the deep tissues than those with the other cooling temperatures except 30 °C (Table 4). At the surface, the lowest Hb was not different from that with the other cooling temperatures except 25 °C (Table 4). The lowest Hbs after cooling at 25 °C were significantly lower than those achieved after 20 °C (*p* = 0.0087; Fig. 2c). At 60 min after cooling at 20 °C, the skin temperature decreased by 0.01 °C. This decrease was within the accuracy of the thermometer used. Each of the other temperatures led to an increase in skin temperature (Table 5). The females were significantly more satisfied with the higher cooling temperatures, e.g., 25 °C or 30 °C (*p* < 0.0001). These results led to the assumption that 20 °C was the optimal cooling temperature.

### Males

In the male participants, cooling at 10 °C led to the greatest reduction of blood flow in the tissues (Fig. 2a). Cooling at 15 °C produced the second greatest reduction. The flow value from cooling at 10 °C was significantly lower (*p* = 0.0043) in the deep tissues and not significantly lower (*p* = 0.254) measured at the surface (Table 1). The 20 °C temperature led to the third biggest decrease in blood flow after cooling. The deep tissue blood flow value after cooling at 20 °C was significantly higher than that achieved after 10 °C (*p* < 0.0001) but not after 15 °C (*p* = 0.0537). Sixty minutes after termination of cooling at 10 °C, the blood flow increased by 15.98 AU measured in the deep tissues and by 15.07 AU at the surface. Cooling at 15 °C led to a smaller increase in the blood flow after 60 min (deep: *p* = 0.899; surface: *p* = 0.2807). There was no significant difference between these values and those achieved after cooling at 10 °C (Table 2). The smallest increase in blood flow and thus reactive hyperaemia was achieved while cooling at 20 °C (Table 2). Cooling at 20 °C led to the greatest reduction in the SO_2_ at the surface and in the deep tissues. Cooling at 20 °C yielded a significantly lower value than the lowest value obtained at 10 °C (deep: *p* = 0.0252; surface: *p* = 0.0013) and the surface value after cooling at 15 °C (deep: *p* = 0.7293; surface: *p* = 0.0426). The Hbs were reduced after cooling at 10 °C, 15 °C and 20 °C; however, there was no significant difference in the values achieved after cooling at each temperature (Table 4). With every cooling temperature, the skin temperatures increased within the same range (Table 5). There were significant differences in the satisfaction values for cooling at 10 °C, 15 °C and 20 °C (10 °C vs. 15 °C: *p* = 0.0072, 15 °C vs. 20 °C: *p* = 0.0131). The males were more comfortable with 20 °C than with the lower cooling temperatures (Table 5). Consequently, the optimal cooling temperature for males should be between 15 °C and 20 °C.

### Limitations

This pilot study recorded cooling effects for a wide range of temperatures. Based on the results, the determination of the optimal cooling temperature for the males was not possible. In future studies, the cooling temperatures should be 15–25 °C with a finer grading than in this study. This could provide more detailed information on the optimal cooling temperature. We are aware that our findings based on exploratory analyses do not provide rigorous evidence of the optimal cooling temperature. Further confirmatory trials are needed before changing clinical practice.

The collective for measurement consisted of volunteers without craniofacial surgery or trauma. The prerequisites of volunteers suffering a surgical trauma could be different from the healthy volunteers. The trauma would lead to inflammation with a higher blood flow in the traumatised area. For this study healthy volunteers were chosen because the physiological reaction to cooling is estimated to be the same in traumatized and heathy volunteers. Additionally, the type of facial trauma was not standardisable. A zygomatic fracture could be more severe and lead to more symptoms of inflammation than another zygomatic fracture. Therefore, the physiological reaction of healthy volunteers was more comparable. The limitation of this study conducted with healthy volunteers was that a reduction of pain could not be measured and included in the determination of the ideal cooling temperature.

The reactive hyperaemia after cooling was not as significant as had been expected. Compared to other studies in the literature that investigated reactive hyperaemia after cooling of sacral skin^19^, it took longer in this study until the reactive hyperaemia occurred. In the study conducted by Liao et al.^19^ the reactive hyperaemia occurred immediately after the cooling stopped while in this study the reactive hyperaemia could be measured after 60 min. Compared with the other study, the measurement period chosen in this study was sufficient. Nevertheless, the reactive hyperaemia could be stronger at later time points. The participants were measured every 15 min for 60 min after cooling. They were required to lie still to prevent the measurements from being influenced by the increase in circulation after exertion. The complete measurement and cooling lasted 120 min. Extending the measurement time would require the participants to lie still for a longer time, and this would increase the data gathering challenges.

## Conclusion

Considering the objective measurements and the subjective opinion of the volunteers, the optimal cooling temperatures for females and males were different. The females’ perceptions of temperature were different from those of males. In addition, there were sex differences in the physiological effects. For females, around 20 °C is an optimal cooling temperature. For males, it is around 15–20 °C, which is slightly cooler. After 60 min, there is only a mild hyperaemia and hyperthermia.

## Data Availability

The data can be accessed in a Figshare repository cited in the references.
